# Intimal angiosarcoma of the thoracic aorta diagnosed by endobronchial ultrasound-guided transbronchial needle aspiration: a case report

**DOI:** 10.1186/s13256-020-02542-2

**Published:** 2020-11-21

**Authors:** Liliana Fernández-Trujillo, Daisy C. Buenaventura, Luz F. Sua

**Affiliations:** 1 Department of Internal Medicine, Pulmonology Service, Interventional Pulmonology, Fundación Valle del Lili, Av. Simón Bolívar. Kra. 98 # 18-49. Torre 6, 4th Floor, Cali, 760032 Colombia; 2 Faculty of Health Sciences, Universidad Icesi, Cali, Colombia; 3 Department of Internal Medicine, Fundación Valle del Lili, Cali, Colombia; 4 Department of Pathology and Laboratory Medicine, Fundación Valle del Lili, Cali, Colombia

**Keywords:** Angiosarcoma, Aorta, Intimal sarcoma, EBUS-TBNA, ROSE

## Abstract

**Background:**

Primary malignant tumors of the aorta are extremely rare. They are frequently located in the abdominal aorta, followed by the thoracic aorta. Sarcomas are the most common histological type. These tumors originate from the middle or intimal layer, the latter being the most common. Symptoms and radiological findings are generally nonspecific. Since their growth is endovascular, embolic phenomena can occur leading to occlusive signs and symptoms.

**Case presentation:**

We describe the case of a 75-year-old Hispanic man, a former tobacco smoker, with a history of pain and epigastric tenderness, dysphagia, and weight loss of approximately 6 kg. A thorax computed tomography scan showed a mass within the posterior mediastinum with poorly defined borders and heterogeneous density, located between thoracic vertebra 5–8, with a size of 78 × 53 × 76 mm, with left main bronchus compression. Endobronchial ultrasound-guided transbronchial needle aspiration was performed; it found an extrinsic posterior compression of the left main bronchus with no endobronchial injury. An intimal angiosarcoma of the thoracic aorta was diagnosed.

**Conclusion:**

Tumors of the aorta are rare and difficult to diagnose; they are a challenge during the diagnosis, since they usually require open surgical procedures. Endobronchial ultrasound-guided transbronchial needle aspiration associated with rapid on-site examination offered, in this case, the possibility of a successful diagnosis, avoiding major procedures. This is the first case reported in the literature of an intimal angiosarcoma of the thoracic aorta diagnosed using endobronchial ultrasound-guided transbronchial needle aspiration.

## Background

Primary malignant tumors of the aorta are extremely rare and have heterogeneous histology. They were first described by Brodowski in 1873 [1]. They are most commonly located in the abdominal aorta, followed by the thoracic aorta. Sarcomas are the most common histological type, which includes intimal angiosarcomas. These tumors usually appear in older men. Their symptoms and radiological findings are generally nonspecific, thus, clinical diagnosis is difficult. Anatomical sites that are difficult to reach for biopsy can lead to a delay in proper identification. Intimal angiosarcomas have endovascular growth; they might cause occlusive signs and symptoms, secondary to embolic phenomena [2]. Usually, diagnosis is confirmed after surgical resection [3]. We present the case of a 75-year-old former tobacco smoker who was diagnosed as having an intimal angiosarcoma by endobronchial ultrasound-guided transbronchial needle aspiration (EBUS-TBNA) with rapid on-site examination (ROSE). EBUS-TBNA is currently the preferred method to diagnose and stage lung cancer because it allows mediastinum evaluation, including extrathoracic metastasis with real-time visualization and sampling. This is the first report of an intimal angiosarcoma of the aorta diagnosed by this method.

## Case presentation

**Fig. 1:**
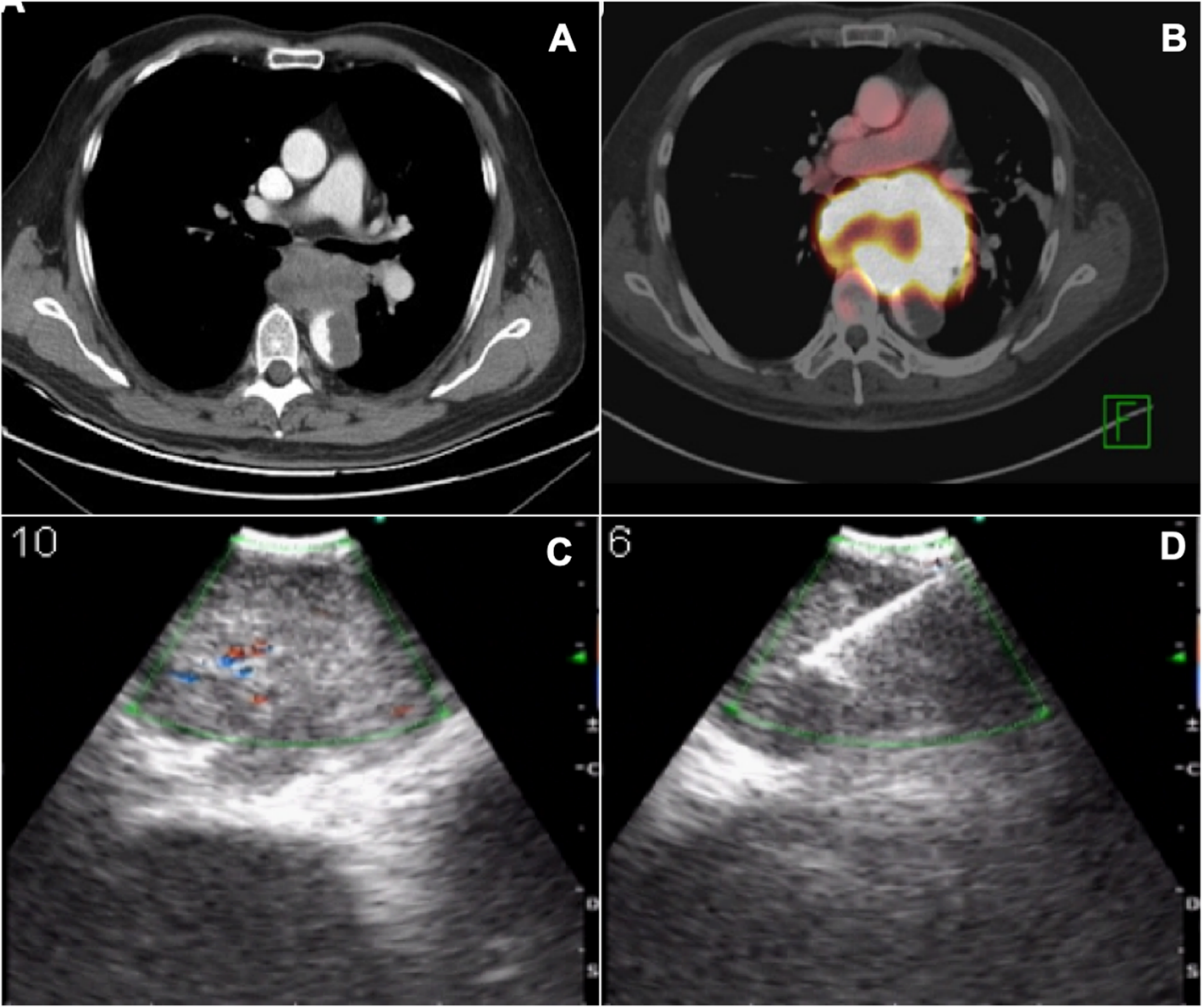
**a** Thorax computed tomography scan showing a mass in the posterior mediastinum that compresses the left main bronchus and infiltrates the aorta with obstruction greater than 50%. **b** Positron emission tomography-computed tomography scan showing hypermetabolic lesion that compresses the left main bronchus and infiltrates the aorta. **c** Endobronchial ultrasound showing a rounded lesion adjacent to the left main bronchus. **d** Image of the real-time fine needle puncture of the tumor for sampling

A 75-year-old Hispanic man, a former tobacco smoker with a history of chronic obstructive pulmonary disease for which he was on inhaled bronchodilators, consulted with a 4-month history of diarrhea, persistent epigastric abdominal pain, dysphagia, and weight loss of approximately 6 kg. During initial examination, his vital signs were normal and his oropharynx unremarkable; no abnormalities were detected in his head and neck or heart auscultation. Respiratory sounds were generally decreased without any other abnormalities. During an abdominal examination, epigastric tenderness was found. His extremities were symmetrical, without edema, and he had normal peripheral pulses. Abdomen ultrasound, colonoscopy, and upper gastrointestinal endoscopy from the out-patient consult were normal. An abdominal computed tomography (CT) scan showed an irregular mass located close to the posterior esophagus wall, causing extrinsic compression, with heterogeneous highlight and infiltration of the descending aorta, which had an intramural thrombus. A thoracic CT scan showed a mass in the posterior mediastinum, with poorly defined borders and heterogeneous density, located between thoracic vertebra 5–8 (T5–T8), with size of 78 × 53 × 76 mm, with left main bronchus compression, posterior displacement of the esophagus without cleavage plane, which surrounded and infiltrated the aorta with an intraluminal thrombus causing a 50% obstruction (Fig. 1a).

**Fig. 2:**
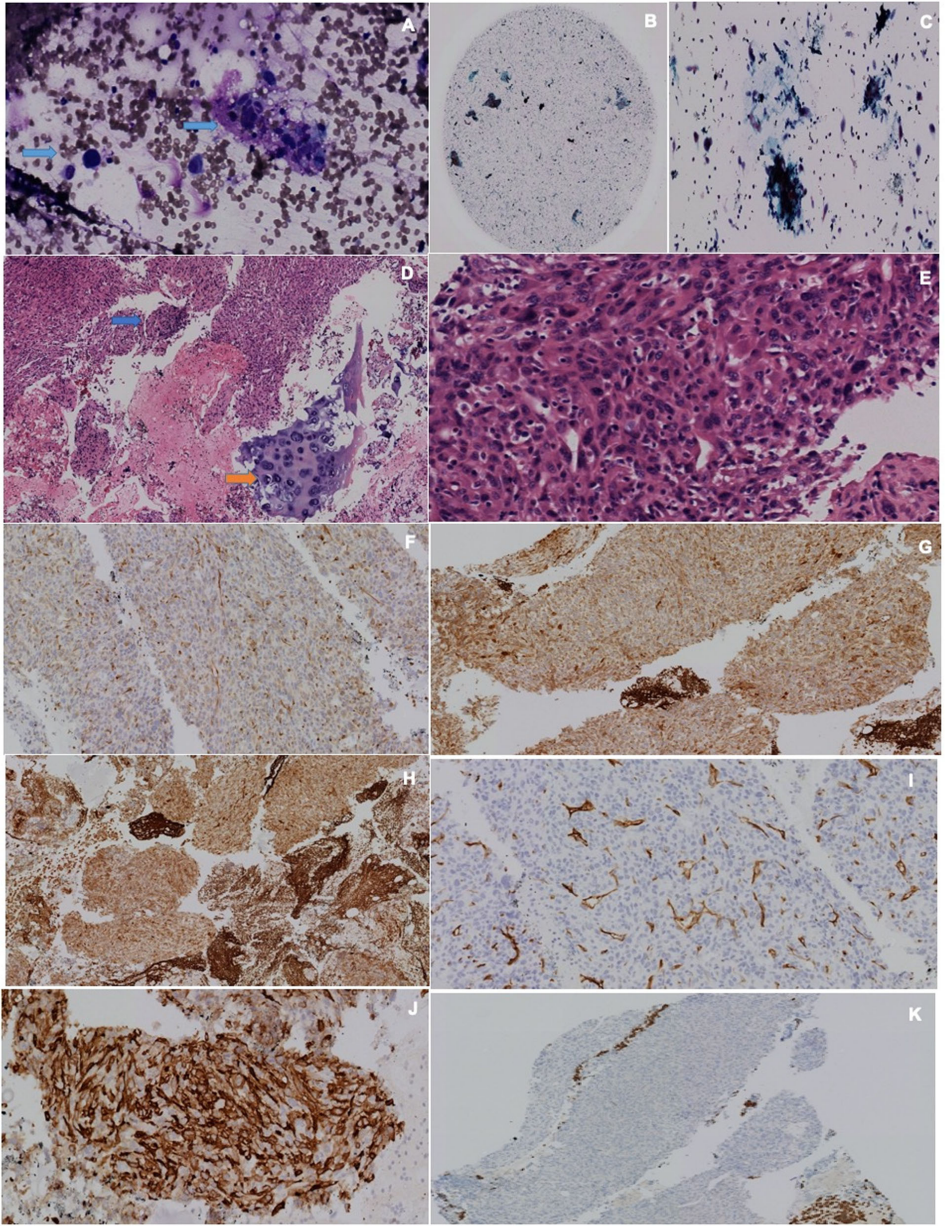
**a** Rapid on-site evaluation. With Diff-Quick staining, large and pleomorphic cells (*blue arrows*) are observed, in the background there are red blood cells. **b**, **c** Liquid-base cytology, which shows some large, pleomorphic cells and respiratory epithelium. Papanicolaou staining. **d** Cell block, hematoxylin and eosin staining. Neoplastic lesion, which shows large and pleomorphic cells (*blue arrow*) with the bronchial wall cartilage (*orange arrow*). **e** Cell block, hematoxylin and eosin staining. Cohesive, large, pleomorphic tumor cells with dense chromatin and up to 22 mitosis on 10× magnification. **f** BCL-2 positive tumor cells. **g** Calponin positive tumor cells. **h** Smooth muscle actin positive tumor cells. **i** CD34 negative tumor cells. **j** CD31 positive tumor cells. **k** CKAE1/AE3 negative tumor cells

A positron emission tomography (PET)/CT scan showed a hypermetabolic mass with malignant features in the posterior mediastinum (Fig. 1b). The mass was heterogeneous with a necrotic center, aortic infiltrations, esophagus and left main bronchus compression, and metastatic hypermetabolic lesions affecting the left suprarenal gland, psoas muscle, obturator muscle, right transverse process of third lumbar vertebra (L3), right ischium, and pubis. After discussing the case with a multidisciplinary team of pulmonologists, thoracic surgeons, and oncologists, we decided to perform an EBUS-TBNA taking into consideration the location of the lesion and with the hypothesis that the primary tumor was inside the bronchi. Although metastatic lesions were more approachable, we decided the best option was to start by studying what we considered was the primary tumor. EBUS-TBNA showed an extrinsic posterior compression of the left main bronchus with no endobronchial injury. Real-time samples through the left main bronchus were performed (Fig. 1c, d). ROSE was conducted to guarantee quality of the sample. Using the Diff-Quick stain, it was confirmed that the sample was positive for malignancy, large pleomorphic cells with a hemorrhagic background were observed (Fig. 2a).

Five passes were needed for a successful sample. Liquid-base cytology was performed, building a block which underwent Papanicolaou staining (Fig. 2b, c). Sample analysis confirmed malignancy due to the presence of large pleomorphic cells with wide eosinophilic cytoplasm, nuclei with anisokaryosis, dense chromatin and nucleolus, atypical mitosis, and 22 normal mitosis at 10× magnification power. The tumor was organized cohesively, with inflammatory mononuclear interposed cells and extensive necrotic areas (Fig. 2d, e). The immunohistochemistry study showed reactivity for BCL-2 proteins, smooth muscle actin, and calponin (Fig. 2f, g), and positivity for smooth muscle actin (Fig. 2h). CD34 and CKAE1/AE3 were negative; CD31 was positive in tumor cells (Fig. 2i, j, k) which meant it was a mesenchymal neoplasia with a high degree of malignancy, classified as an intimal angiosarcoma.

Angiosarcomas are classified histopathologically as intimal or mural subtypes, which is clinically relevant since they have different behaviors and prognoses. Intimal sarcoma is polypoid, involves the intima of blood vessels, and grows in the direction of the aortic lumen; it frequently generates occlusion and embolic phenomena. Mural angiosarcoma originates in the media or adventitia and it predominantly grows toward adjacent structures. Immunohistochemistry is essential to confirm the diagnosis. Positivity of CD31 is of great value for the confirmation of an angiosarcoma. Due to the extension of the tumor, no surgical treatment was considered. The oncology team proposed to do two consecutive cycles of chemotherapy: doxorubicin 10 mg/intravenous for 3 days, Isofosfamide (ifosfamide) 1000 mg/intravenous for 4 days, and mesna 400 mg for 4 days was given. After the second course of chemotherapy there was no response to treatment and our patient presented multiple adverse effects, mainly intense abdominal pain and dysphagia. The palliative care team was consulted, who decided to begin management with analgesia; they supported our patient and his family in the process of bereavement. One week later, our patient died.

## Discussion and conclusions

This is the first case of an intimal aorta angiosarcoma diagnosed by EBUS-TBNA, which in this case was the only option, anatomically, to approach the lesion and to guarantee a timely diagnosis without any further interventions. Although the literature supports that core biopsies have a better sensitivity and a lesser risk of localized spread compared to needle aspiration, we believed the primary tumor was inside the bronchi and the rest of the alterations corresponded to metastasis, being irresectable, which placed EBUS-TBNA as the optimal technique considering it is minimally invasive. Furthermore, the mass strongly adhered to the aorta and it was in an anatomical location that was very difficult to reach by open surgery, as considered by the thoracic surgeon who was in agreement with the less invasive approach. EBUS-TBNA is the preferred method to diagnose and stage lung cancer. This technique allows mediastinum evaluation, including extrathoracic metastasis with real-time visualization and sampling [4]. Complications are reported in less than 1% of cases; the most common complications are: vascular injury, minor bleeding, pneumothorax, pneumomediastinum, mediastinitis and, very rarely, fatal hemorrhage [5]. When in station 5, which includes ganglia in the aortic-pulmonary vein, needle biopsy is preferred over a surgical approach with a cervical mediastinoscopy or anterior mediastinotomy, since vascular structures must be crossed to reach them during EBUS-TBNA [6].

In some hospitals, endobronchial ultrasound-guided transvascular needle aspiration (EBUS-TVNA) is performed without complications, when sites for biopsy are not accessible using traditional endobronchial ultrasound, such as the aortopulmonary window [7, 8]. In this case, transbronchial puncture was done without any complications. The importance of ROSE must be highlighted in the performance of EBUS-TBNA and EBUS-TVNA, in which the number of passes and punctures through the vessels is reduced [9].

## Data Availability

All data and materials are available for sharing if needed.

## Abbreviations

EBUS-TBNA: Endobronchial ultrasound-guided transbronchial needle aspiration
ROSE: Rapid on-site examination
CT: Computed tomography
T5–T8: Thoracic vertebra 5–8
PET: Positron emission tomography
EBUS-TVNA: Endobronchial ultrasound-guided transvascular needle aspiration
L3: Third lumbar vertebra
